# The transition of legal status among Korean immigrants in the United States: immigration story, challenges, and mental health

**DOI:** 10.1016/j.jmh.2025.100359

**Published:** 2025-09-20

**Authors:** Chulwoo Park, Airi Irene Trisnadi

**Affiliations:** a Department of Sociology, Anthropology, and Public Health, University of Maryland, Baltimore County, Baltimore, MD 21250, USA; b Department of Psychology, San José State University, San Jose, CA 95192, USA

**Keywords:** Immigration, Korean immigrants, Legal status, Mental health

## Abstract

**Background:**

With an increase in the Asian immigrant population, Koreans are among the top five Asian immigrants in the U.S. When immigrants move to the U.S., many encounter challenges related to obtaining, transitioning, or maintaining their legal status. Temporary immigration visa, including H1-B working visas and F-1 student visas, allows immigrants to move to or remain in the U.S. Immigrants planning to stay in the U.S. are required to renew their working visa or obtain a green card or U.S. citizenship. While existing studies focused on the challenges of obtaining legal status, there is little emphasis on the immigration experiences of Korean immigrants. This study aimed to examine the challenges faced by Korean immigrants in obtaining primary legal status when coming to the U.S. and transitioning to their current legal status after moving to the U.S. Furthermore, this study assessed the relationship of change in legal status with experiences of racism and discrimination and immigrant mental health.

**Methods:**

A qualitative study was conducted with 24 participants through an online survey for demographic data collection, followed by semi-structured in-depth interviews. Participants consisted of Korean immigrants who attended a Korean Christian church in the San Francisco Bay Area. Interviews were conducted between March 2023 and July 2023.

**Results:**

The majority of immigrants came to the U.S. with an F-1 student visa and currently hold an H1-B visa, green card, or U.S. citizenship. While immigrants faced relatively few challenges in obtaining their primary legal status before coming to the U.S., many struggled with the transition from an F-1 student visa to an H1-B working visa. Common challenges included finding company sponsorship and being selected in the visa lottery. In addition, immigrants experienced an additional set of challenges through racism or discrimination and cultural shocks upon moving to the U.S., which contributed to their mental health.

**Conclusions:**

This study provided a new perspective on the challenges encountered by Korean immigrants, focusing on the differences in obtaining their primary legal status and transitioning to their current legal status. We showed the importance of assessing the effects of change in legal status on the psychological well-being of Korean immigrants by looking into the immigrants' experiences of racism and discrimination and analyzing the immigrants' mental inconsistencies of their expectations versus reality. These findings open the way for future research on Asian immigrants in the U.S. and contribute to the development of U.S. immigration policies.

## 1 Introduction

### 1.1 Korean immigrants' legal status and mental health

Since the early 20th century, a rapid increase in Asian immigrant populations in the U.S. has been observed. In 2022, approximately 46 million individuals living in the U.S. were foreign-born with nearly 31 percent of immigrants making up the Asian population (U.S. Census Bureau, 2022). Among the Asian population, Chinese descent was the largest ethnic group with approximately 5.2 million individuals, followed by Indian, Filipino, Vietnamese, and Korean (U.S. Department of Commerce, 2023). The Korean population falls within the top five ethnic groups in the U.S. (Esterline and Batalova, 2022), yet there is limited emphasis on the immigration patterns of Korean immigrants. As immigrants move to the U.S., many face concerns and issues when attempting to obtain, change, or maintain their legal status (Jacobs, 2019; Kim, 2022).

When moving to the U.S., immigrants are required to complete a visa application process to obtain legal status to stay in the U.S. from the U.S. Citizenship and Immigration Services (USCIS). Immigrant visas are provided based on family connections, employment or education, and other special immigrant categories (U.S. Department of State - Bureau of Consular Affairs, 2024). These visas include but are not limited to, H1-B working visa, F-1 student visa, J-1 exchange student visa, and Green Card. According to the Office of Homeland Security Statistics of 2022, the two highest categories of admission were student or exchange visitors and temporary workers, excluding tourists and business travelers (Office of Homeland Security Statistics, 2023). Legal working and student visas allow for temporary stay in the U.S., often requiring sequential renewal to maintain or obtain new legal status. Maintaining a working visa involves competitive application processes. According to U.S. Citizenship and Immigration Services, application submissions for H1-B visas exceed the cap limit every year (U.S. Citizenship and Immigration Services, 2024a). Therefore, the USCIS conducts an annual lottery in March to randomly select applications to be processed. In addition, student immigrants with F-1 visas are eligible for OPT for up to 12 months where they are able to work for a year upon graduating from college. However, after 12 months, students are either required to apply for an H1-B visa or find a company for sponsorship to continue to legally stay in the U.S.

After 5 years as lawful permanent residents (LPR), or Green Card holders, individuals are eligible for naturalization. Naturalization involves the process of obtaining U.S. citizenship as an LPR who has fulfilled the requirements of the Immigration and Nationality Act (U.S. Citizenship and Immigration Services, 2020a). According to the U.S. Department of Homeland Security, nearly 290,000 individuals were naturalized in 2022, indicating a 21 percent increase from the year 2021(U.S. Department of Homeland Security, 2023). Among those naturalized in 2022, 14,880 immigrants had originated from South Korea with California being the leading state of Korean American residence with approximately 30 percent residing in California, followed by New York, New Jersey, Georgia, and Texas (U.S. Department of Homeland Security, 2022).

Previous research on immigrant legal status focused on comparing the psychological well-being of immigrants across different legal statuses (H1-B, F-1, green card, etc.) (Brabeck and Sibley, 2016; Jacobs, 2019; Kim, 2022; Singh et al., 2017). As foreigners immigrated to the U.S., psychological consequences related to racism and discrimination were detrimental to the adjustment to the new environment. In particular, the Korean population perceives higher levels of racial discrimination compared to other Asian ethnic subgroups (Cho et al., 2022; Ruiz et al., 2023). Discrimination against Asian Americans commonly arises from language barriers, cultural differences, and diverse ethnic backgrounds, which may lead to mental health concerns including lower self-esteem and a diminished sense of community connection (Alegría et al., 2017; Nadimpalli et al., 2016; Yoo et al., 2009).

While many studies supported the psychological effects of maintaining different legal statuses on immigrants, the challenges involved during the process of changing from one legal status to another have not been extensively researched. Examining the differences in challenges when obtaining legal status before and after coming to the U.S. reveals specific struggles unique to immigrants based on their status and changes in visa challenges over time. The purpose of this study was to analyze the challenges among the Korean immigrant population when obtaining primary legal status when coming to the U.S. and when transitioning to their current legal status after moving to the U.S. including racism and discrimination, and its relationship to their mental health.

### 1.2 Theoretical frameworks and Korean immigrant experiences

Incorporating established theoretical frameworks such as Berry (1997)'s acculturation model and Massey et al. (1993)'s theories of international migration provides a strong foundation for understanding the diverse experiences of Korean immigrants. Berry's model explains how immigrants balance preserving their cultural identity while adapting to the host society, highlighting the psychological stress and coping mechanisms that arise during acculturation. Massey et al.'s migration theory explores the structural forces driving migration and the social, economic, and political factors that shape immigrant adaptation. Additionally, Portes and Zhou (1993)'s segmented assimilation theory offers insights into how different social environments influence immigrant integration and long-term well-being. Gee and Ford (2011)'s theory of structural racism and health inequities expands on this by emphasizing how institutional discrimination and broader societal structures perpetuate health disparities among racial minorities, including Asian immigrants. By grounding the study in these frameworks, the research can better contextualize the personal experiences of Korean immigrants within broader patterns of migration and acculturation, contributing to a deeper analysis of cultural adjustment, discrimination, and health disparities.

## 2 Material and methods

### 2.1 Study design

Qualitative in-depth interviews were conducted with a total of 24 participants to analyze the relationship between the change in legal status and the impact on immigrant mental health. The inclusion criteria were as follows: 1) participants' parents must be Korean (included even if parents' current nationality is not South Korean), 2) must be 18 years of age or older, 3) must be attending church, and 4) must be able to read and speak in English. We administered an online survey using Qualtrics^XM^ (Qualtrics International Inc., Provo, UT) to collect demographic information. Detailed responses were obtained through the in-depth interviews. This study focuses on the narratives of Korean immigrants regarding their journey immigrating to the U.S.

### 2.2 Participant selection and data

Participants were selected based on purposive (*N* = 17, 70.83 %), random (*N* = 3, 12.5 %), and snowball (*N* = 4, 16.67 %) samplings. Individuals were recruited through group leaders at a local Korean church for the convenience of conducting in-person interviews. We asked church group leaders to distribute the Qualtrics survey link to members in their groups. Within the survey, we assessed their willingness to participate in an in-depth interview. Individuals expressing interest in the in-depth interview received an invitation and confirmation emails. A total of 24 participants consisting of male (*N* = 12) and female (*N* = 12) immigrants completed the in-depth interview.

### 2.3 Interview setting

Semi-structured in-depth interviews took place at a church located in Mountain View, California. A total of 23 interviews were conducted in-person with one interview conducted online via Zoom (Zoom Video Communications, Inc., San Jose, USA) to accommodate the participant's accessibility. The interviews were carried out by the principal investigator (PI) and the researchers of the study. For the majority of interviews, there was a primary interviewer and an assistant interviewer to ensure the quality of the participant responses. The interviews took place between March 2023 and July 2023.

### 2.4 Data collection and instrument

Demographic information (Table 1) was collected through the distribution of the Qualtrics survey while detailed responses were gathered through the in-depth interviews.

**Table 1:** Demographic characteristics of participants.

	N (percent)
Gender	
Male	12 (50)
Female	12 (50)
Age (in years)	
20–30	5 (20.83)
31–40	13 (54.17)
41–50	6 (25)
Living location	
Large city or small city/town	7 (29.17)
Suburb near a large city	16 (66.67)
Do not know	1 (4.17)
Highest level of education	
Two-year associate degree from a college, university, or community college	1 (4.35)
Four-year bachelor's degree from a college or university	8 (34.78)
Some postgraduate or professional schooling after graduating college, but no postgraduate degree	1 (4.35)
Postgraduate or professional degree including master's, doctorate, medical, or law degree	12 (52.17)
Place of employment	
A for-profit private company, business, or individual	19 (82.61)
Other (non-profit, educational institution, self-employed)	3 (13.04)
Not currently employed	1 (4.35)
Having an children	
Yes	2 (8.33)
No	22 (91.67)
Living alone	
Yes	11 (45.83)
No	13 (54.17)
Total household income	
$35,001 - $50,000	2 (8.33)
$50,001 - $150,000	5 (20.83)
$150,001 or more	13 (54.17)
Do not wish to answer	4 (16.67)
Marital status	
Single/Never been married	20 (83.33)
Married	3 (12.5)
Separated/Divorced/Widowed	1 (4.17)
Visa status when first coming to the U.S.	
Already had a Green Card	2 (8.33)
Born with U.S. citizenship	2 (8.33)
H1-B visa	0 (0)
F-1 visa	12 (50)
Other	8 (33.33)
Current visa status	
Have obtained a Green Card	7 (29.17)
Have obtained U.S. citizenship	10 (41.67)
H1-B visa	4 (16.67)
F-1 visa	2 (8.33)
Other	1 (4.17)

Regarding saturation, we observed consistent thematic recurrence during the interviews. By the 21st interview, no new major themes emerged, suggesting that thematic saturation was achieved. However, we proceeded with three additional interviews to confirm this and ensure the robustness of our data. Participants were scheduled for a semi-structured interview with an average duration of 59 min (ranging from 26 min to 95 min). Interviews were recorded through the use of audio recordings. Recordings were captured using voice recording devices positioned directly in front of participants during each interview. These recordings were then uploaded and transcribed using Otteri.ai (Otter.ai, Inc., Mountain View, USA), a transcription application software. Subsequently, researchers manually refined the transcriptions to ensure accuracy. Participant demographic data and interview responses were initially documented using their actual names, which were later anonymized through the assignment of pseudonyms to protect participant confidentiality (Table 2).

We focused on seven questions related to the purpose of this study as follows:  

*Before coming to the U.S.*

Q1. Were you mentally prepared to leave your home country? If yes, what makes you think you were prepared?
Q2. What was your initial expectations of coming the U.S.? Was it different than expected? If so, how?
Q3. Can you share any difficulties fulfilling the requirements or preparing the documents for gaining legal status when first coming to the U.S.? If you faced difficulties of first coming to the U.S., how did you deal with it? Did you get any support?

*After coming to the U.S.*

Q4. What cultural shock have you experienced when you first came to the U.S.? What cultural shock you still have?
Q5. Have you experienced any prejudice or discrimination in the U.S.?
  1) Due to English language proficiency
  2) Due to race/ethnicity
  3) Due to different culture: Korean culture vs American culture
Q6. Have you been experiencing any challenges and difficulties when pursuing the legal status that you would like to obtain?
Q7. How would you describe your mental health while pursuing F-1 visa, H1B visa, or the visa that you came with?

**Table 2:** Individual characteristics from participants.

Participant Number	Participant Pseudonym	Age	Gender	Visa when first coming to the U.S.	Current Visa status
1	Sophia	42	Female	B-1 or B-2	US citizenship
2	Marc	32	Male	F-1	Green card
3	Joy	34	Female	Green card	US citizenship
4	Danielle	32	Female	F-1	Green card
5	Jessica	32	Female	US citizenship	US citizenship
6	Shawn	39	Male	J-1	US citizenship
7	Josh	35	Male	F-1	Green card
8	Jeffery	35	Male	F-1	US citizenship
9	Jeremy	32	Male	F-2	US citizenship
10	Sarah	42	Female	B-1 or B-2	Green card
11	Yolanda	30	Female	F-1	H1-B
12	Clara	46	Female	Green card	US citizenship
13	Sienna	36	Female	F-1	Green card
14	Yvonne	44	Female	J-1	Green card
15	Lisa	33	Female	F-1	F-1
16	Grace	42	Female	J-1	US citizenship
17	Derek	33	Male	F-1	US citizenship
18	Donald	35	Male	US citizenship	US citizenship
19	Jack	29	Male	F-1	H1-B
20	Michael	35	Male	F-1	F-1
21	Yasmine	25	Female	F-1	H1-B
22	Jerome	45	Male	F-1	H1-B
23	Clay	30	Male	J-1	O-1
24	Wesley	20	Male	F-4	Green card

### 2.5 Data analysis

**Fig. 1:**
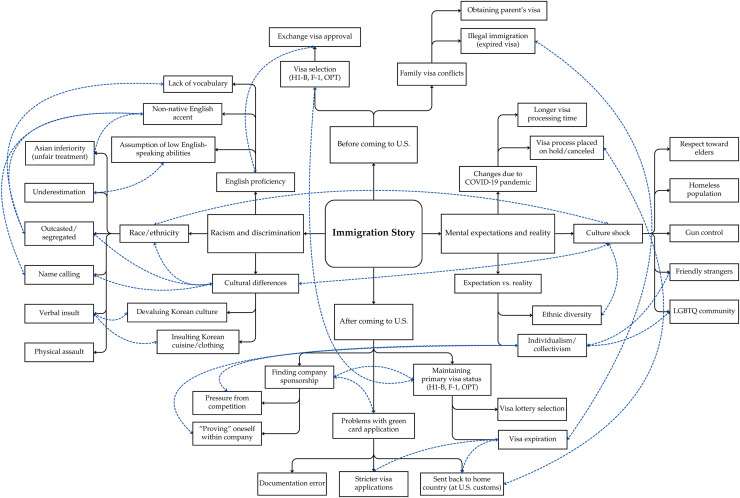
Interrelationship between codes and sub-codes.

Using qualitative analysis, the research team examined all interview transcripts and collectively identified themes derived from the interview questions pertaining to participants' immigration stories. Researchers took note of events documented in each transcript and developed a set of codes through inductive coding, which were reviewed by other team members. Fig. 1 illustrates the interrelationship between codes and sub-codes related to immigration challenges before and after coming to the U.S., racism and discrimination, and the inconsistency between mental expectations and reality. Solid black lines represent the codes and categories for each sub-theme, while dotted blue lines connect codes that were commonly mentioned together in the in-depth interviews.

### 2.6 Ethical considerations

This study has been carried out in accordance with the guidelines of the Institutional Review Board (IRB) for the online survey and in-depth interview. Consent notices were provided to participants at the start of the online survey and at the beginning of the interview. Participants were informed of the confidentiality of their identity with the use of pseudonyms. For the interview conducted online, the participant was informed of the option to turn off their video in Zoom. To prevent identification, participants are identified by their pseudonyms within the manuscript and in the demographic table (Table 2). Pseudonyms were assigned using an online random pseudonym generator.

## 3 Results

Participants were asked the challenges they faced in obtaining legal status before and after coming to the U.S., experiences of racism and discrimination, and inconsistencies of their expectations and reality of the U.S. Their experiences (i.e., challenges, discrimination, and expectations) were divided into the following four categories: 1) challenges in obtaining primary legal status before coming to the U.S., 2) racism and discrimination after coming to the U.S. 3) challenges in transitioning to their current legal status after coming to the U.S., and 4) the differences in expectations and reality of the U.S.

### 3.1 Before coming to the U.S., immigrants faced challenges related to family visa conflicts and difficulty in visa selection when obtaining primary legal status

Korean immigrants encountered a variety of difficulties in obtaining legal status before coming to the U.S. Common challenges included difficulty obtaining visas through visa selection and family visa conflicts. The immigrants' primary legal status consisted of an F-1 student visa, J-1 exchange visa, and B-1 or B-2 visitor visa. The challenges participants experienced in obtaining legal status before coming to the U.S. were extracted and analyzed below.

#### 3.1.1 Visa selection (F-1, J-1 visa)

##### 3.1.1.1 Exchange visa approval

To study abroad, immigrants must complete and adhere to the eligibility requirements from the government of the country of origin as well as the requirements given from the school to which the immigrants apply as exchange students. However, some international institutions restrict students from attending certain schools in the U.S. Josh shared his experience of getting denied an F-1 visa because the high school he applied to was not approved by the Korean government. Even when applying for university, he was worried that the F-1 visa that was not approved by his high school would prevent him from being accepted:

For the F-1 visa, I tried to go to Texas for [an] exchange student, but my [high] school was not approved by [the] Korean government at that time. So I got denied twice, two years in a row… . I was very worried. Even for university, I [was] already rejected twice and I thought I might not be able to attend the university because of the visa. (Josh, age 35, Male, F-1 visa)

#### 3.1.2 Family visa conflicts

##### 3.1.2.1 Obtaining parent's visa

Second-generation immigrants often rely on their parents' visas to obtain legal status. Young children who came to the U.S. with their parents often acquired the same visa as their parents. Sophia shared how she was unable to get a green card when she was younger because her parents experienced conflicts in gaining legal status. Her mother had to find a church that would help her apply for a green card and had to be given a position in the church ministry in addition to playing the piano to be eligible for a green card for employment:

My mom majored in piano… . It was a struggle because we were told that you cannot apply for just playing [the] piano… so my mom was given the title of director for the church's ministry… in order to gain legal status here. (Sophia, age 42, Female, B-1 or B-2 visa)

##### 3.1.2.2 Illegal immigration (expired visa)

The status of the immigrant parents' visa tends to affect the legal status of second-generation immigrants. When parents overstay their visa expiration date, they are taken away from their visa, consequently causing their children to lose legal status as well. When Jeremy was younger, his mother came to the U.S. with an F-1 visa and himself with an F-2 visa. An F-2 visa is a temporary visa provided for immediate family members of an F-1 student visa holder (U.S. Citizenship and Immigration Services, 2023). Even after his mother's visa expired, they continued to stay in the U.S., making Jeremy's legal status invalid:

My mother decided to come here on a student visa [and] there was a period of time where we were basically illegal immigrants… . [because] she overstayed her visa. (Jeremy, age 32, Male, F-2 visa)

### 3.2 After coming to the U.S., immigrants often faced challenges in obtaining sponsorship, problems with visa application processes, and changes due to the COVID-19 pandemic when transitioning to their current legal status

In addition to the difficulties immigrants faced when obtaining legal status before coming to the U.S., Korean immigrants encountered a variety of problems after coming to the U.S. Massey et al.'s theory of international migration highlights how structural forces influence immigrants' experiences, including the challenges they face during the immigration process. Common challenges immigrants faced in obtaining current legal status were problems maintaining or changing primary legal status, difficulties obtaining company sponsorship, problems with visa application process, and facing changes to the visa process due to COVID-19 pandemic. The immigrants' current legal status mainly consisted of having U.S. citizenship, green card, H1-B working visa, or F-1 student visa. The challenges in obtaining their current visa status were extracted and analyzed below.

#### 3.2.1 Maintaining/changing primary visa status (H1-B, F-1, OPT)

##### 3.2.1.1 Visa lottery selection

When obtaining an H1-B, F-1, or OPT visa, immigrants are placed in a lottery system and selected from a competitive pool of applicants (U.S. Citizenship and Immigration Services, 2024a). Due to an increase in visa applications, the USCIS annually approves only a select number of applications. For immigrants desperate to obtain a visa status, the visa selection process led to stress and anxiety as winning the lottery was the only option that allowed them to legally stay in the U.S.:

Even H1-B, I tried two times. I didn't get selected for both of the times so that's kind of heartbreaking. (Michael, age 35, Male, F-1 visa)

For F-1 visa students to obtain an H1-B visa, immigrants are required to apply to the H1-B lottery system (U.S. Citizenship and Immigration Services, 2024a). Oftentimes, immigrants were not selected on their initial attempt and had to reapply in consecutive years. Marc came to the U.S. with a student visa and upon graduation, obtained an H1-B working visa. While he was still on an F-1 visa, he worked at a company and filed to convert his visa to a working visa. However, due to the competitive selection process, it took him two years to officially obtain the H1-B visa:

I had a total [of] 29 months to work [and] every year, you could file for… H1-B visa, but you might not get selected because… there's only limited quotas for visa. I was trying for two years in a row and it didn't work out. (Marc, age 32, Male, F-1 visa)

##### 3.2.1.2 Visa expiration

Before the legal visa expires, immigrants planning to stay in the U.S. are required to reapply to renew their visa. However, the application approval process may take a long time, which at times exceeds the visa expiration date. Participants expressed concerns about meeting the visa deadline while waiting for visa approval to renew or change their visa status. Yolanda shared her stressful experience of waiting for her law firm's approval while she was left uncertain of whether she is able to stay in the U.S. or would have to leave to go back to Korea:

Next March is [the] expiration date so if I don't get approved for my firm [(law firm)] within the couple months, I'll have to leave the country… . It's stressful, for sure. (Yolanda, age 30, Female, F-1 visa)

Even participants with full-time jobs on H1-B or OPT working visas expressed concerns as their visas were close to expiring. After Danielle obtained a working visa and found a job following her studies on an F-1 student visa, she became worried about having to renew her working visa in order to remain in the U.S.:

I got another job [at a] preschool… but I can only work for seven, eight months because my work permit is going [to] expire… . I was very nervous because I need to leave this country as soon as my visa expire. (Danielle, age 32, Female, F-1 visa).

#### 3.2.2 Finding company sponsorship

##### 3.2.2.1 Pressure from competition

To acquire a working visa or green card, immigrants may obtain a company sponsorship (U.S. Citizenship and Immigration Services, 2022, 2024b). However, obtaining sponsorship involves competing to make oneself stand out. In order to obtain a working visa, participants had to find alternative options to prove to the USCIS that they had a reason for staying in the U.S. Lisa studied music as a college undergraduate and needed proof of her talent for piano from competition judges. Lisa shared her experience of struggling to find a recital hall within a short period of time as the majority of recital halls were already booked:

I need to prove myself how I'm.. [a] good musician… . I have my career and the prizes, but my lawyer wanted proof from my competition's judges… . [The] problem is I cannot find [a] good recital hall… . so [it] is really hard. (Lisa, age 33, Female, F-1 visa)

F-1 student visa immigrants considered obtaining company sponsorship as a top priority alternative. Participants expressed the urgency they experienced to get sponsorship as their visas were nearing expiration. Many immigrants were not employed at a company and struggled to gain social connections to receive sponsorship. Danielle shared her feelings of anxiety because finding a company that is willing to sponsor her was the only option for her to remain in the U.S.:

I got job in preschool but I can only work for seven, eight months because my work permit is going [to] expire… . I was very nervous because I need to leave this country as soon as my visa expires so I need to get some sponsorship to get my legal status to remain or to get a green card or at least H1-B. (Danielle, age 32, Female, F-1 visa)

##### 3.2.2.2 “Proving” oneself within company

Korean immigrants felt pressure from the need to “prove” themselves to a company to obtain sponsorship. Participants shared the struggles they experienced when obtaining an H1-B working visa or green card through a company. Although many were relieved to find company sponsorship, they felt pressure to give progress that exceeds the average for them to “prove” to the company that they are worthy of continuing employment with the company and staying in the U.S. Marc mentioned that he did not want to be seen as a “mediocre,” and after years of working, his company had decided to sponsor him for a green card:

I started working, and they're [(the company)] like, “Yeah, sure, we'll sponsor you for the H-1 visa.” I worked hard, not just because it was my job but also, I felt like I needed to prove myself… . One, because they were sponsoring me and two, because I didn't want to be seen as [a] mediocre worker… . I worked pretty hard and… after working [for] three years, they decided to sponsor for the green card. (Marc, age 32, Male, F-1 visa)

#### 3.2.3 Problems with visa application

##### 3.2.3.1 Documentation error

Immigrants experienced documentation problems that delayed their visa application processes. Clara explained an issue she faced when trying to obtain U.S. citizenship regarding the documentation of her name. Her Korean parents had documented her Korean name with different spellings, making the verification process slow. She had to publically announce that she was changing her name and submit proof to verify her name to court:

I had many different spellings of my name so when I was going to get my citizenship, I needed to verify all the names that I used… . I needed to legally put an advertising out that I'm changing my name back to the old [name]… . and submit it to the court… . That was very hard… . [and] was a hassle. (Clara, age 46, Female, Green Card)

Immigrants have the option of hiring an attorney to guide their application and interview processes for obtaining a green card or permanent resident status. While working with a law firm may ensure the fulfillment of requirements, several immigrants experienced conflicts with the law firm regarding documentation errors and longer visa processing times. Jerome shared his experience of having problems with the law firm which interfered with his green card application. He conveyed the mental weight he experienced in resolving the issue as he was not mentally prepared to leave the U.S.:

During the first green card application, there was some mistake made by the law firm. And I had two choices, just go with the application or withdraw and reapply… . The process was pretty painful because I was not prepared to go back to South Korea yet. (Jerome, age 45, Male, F-1 visa)

##### 3.2.3.2 Stricter visa applications

Following the 2016 presidential election, the Trump administration implemented the Reforming American Immigration for a Strong Economy (RAISE) Act in 2017, which aimed to reduce legal immigration to the U.S. by 50 percent through the cutback of green cards issued (Gelatt, 2017). Immigration policies became strict as described by Jerome. He mentioned that the USCIS had been very detailed and precise with the application review as he applied for a green card which extended the application process:

I was in the green card process. Due to the Trump administration… the USCIS became a lot stricter so it took a long way. They meticulously looked into all the application so they became stricter in the review process. (Jerome, age 45, Male, F-1 visa)

##### 3.2.3.3 Sent back to home country (at U.S. customs)

Sienna shared her experience of being sent back to Korea at the U.S. customs. During her green card application process, she returned to Korea to attend her friend's wedding. Upon re-entering the U.S. with her visitor visa, the customs officer questioned her about the purpose of immigrating to the U.S. After Sienna explained her intention to get married in the U.S., the officer took her to the secondary screening process where she was sent back to Korea:

I went to Korea and [when] coming back, at the customs interview… . The officer asked, “What's your purpose for coming into U.S.?”… . I was so naive. I was like, “I got married.”… . Then he sent me to the secondary process… where the deportees go. (Sienna, age 36, Female, F-1 visa)

#### 3.2.4 Changes due to COVID-19 pandemic

##### 3.2.4.1 Longer visa processing time

Due to the COVID-19 pandemic, changes were implemented to immigration regulations and policies. With the lockdown protocols in place, many workers faced layoffs, leading to an increase in visa applications (U.S. Citizenship and Immigration Services, 2020b). Immigrant workers seeking to remain in the U.S. were required to find new employment with a working visa or obtain a green card. Michael experienced a long processing time for his green card due to the major layoffs:

The green card process is still really slow. The reason [is] because there was a massive layoff [a] few months ago… . A lot of people are getting audited… . . The green card process is pretty tough right now, even for H1-B. (Michael, age 35, Male, F-1 visa)

##### 3.2.4.2 Visa process placed on hold/canceled

During the height of the COVID-19 pandemic, the government faced changes in visa procedures, leading to the cancellation of in-person interviews. As a result of the changes, many immigrants including Josh experienced a delay in their visa applications. Josh expressed his concern when his interview for his green card was canceled as the entire application process was unclear at the time. If his application had not been approved, he would have had to return to Korea:

When COVID broke out, my interview got canceled… . [If] I don't get approved… . I have to go back to Korea. Nothing was very clear at that time. People were all worried about what's going to happen. (Josh, age 35, Male, F-1 visa)

### 3.3 Immigrants experienced racism and discrimination regarding language proficiency, race and ethnicity, and cultural differences after moving to the U.S.

After moving to the U.S., Korean immigrants faced racism and discrimination due to their language proficiency, race and ethnicity, and cultural differences. These experiences align with Gee and Ford's theory of structural racism, which highlights how societal structures contribute to inequalities faced by racial minorities. Particularly for immigrants residing in states with a predominantly White population, they were more prone to encounter discrimination. The various forms of discrimination and the different ways in which they impacted immigrants were categorized and examined below.

#### 3.3.1 English proficiency

##### 3.3.1.1 Non-native English accent/pronunciation

For the majority of immigrants, their first language is not English, making it difficult for them to communicate with the local community. Having a non-native English accent made immigrants feel segregated from the other native speakers, and in some cases, be taken advantage of. Jeremy shared his experience of being an “easy target” for being discriminated against and being blamed for:

Even if you speak the same language, if your accent is different, people will make you an easy target for the mass crowd to pick out and blame or make fun of. (Jeremy, age, 32, Male, F-2 visa)

In addition, Derek shared similar experiences of having difficulties building relationships with other classmates as many students disliked and occasionally made fun of him for not being able to fluently speak English:

When I was in middle school, there [was] one kid who hated me because I didn't really speak English… . There are always people like that… who try to make fun of it when you can't speak English. (Derek, age 33, Male, F-1 visa)

##### 3.3.1.2 Lack of vocabulary

Language barrier was a challenge for immigrants as they faced difficulties expressing themselves and communicating effectively with the local community due to their lack of vocabulary. Furthermore, several participants faced discrimination based on their English language proficiency. Jeffery explained how his middle school teachers were “pretty outrageous” as they kicked him out of class for not being able to answer a question:

I was in the class and I didn't speak English, right? So he [Jeffery's history teacher] was like, “Oh, you're not gonna answer my question? You should get out of the class.” [And] he kicked me out of the class. (Jeffery, age, 35, Male, F-1 visa)

##### 3.3.1.3 Assumption of low English speaking abilities

As Korean immigrants, participants shared their experiences of being perceived and treated differently by others based on their appearances. Many local people assumed they had low English speaking abilities because of their race as Asians. When Clara worked at the front desk of a hotel in Las Vegas, a family of white customers spoke to her slowly and clearly. Although she claims that the family's intentions may had been different, Clara felt as if they were mocking her for not being able to speak English as an Asian receptionist:

They just automatically assume that I don't speak English… . They'll be like, “I need to check in” (*in slow speed*) and I'll be like, “You don't need to speak that slow. I can speak English.” (Clara, age 46, Female, Green Card)

#### 3.3.2 Race/ethnicity

##### 3.3.2.1 Asian inferiority (unfair treatment)

For immigrants who resided in states with predominantly White communities, the concept of Asian inferiority was more apparent. Derek had immigrated to Indiana for work, which is considered one of the states where the Asian community is seen as the minority. He mentioned that one of his co-workers had the opportunity to be promoted to a higher position, which Derek felt was due to a limitation from being Asian:

[At] my company, I can tell that I have [a] limit because I'm Asian… . My coworker's wife could go higher than me even though we [have] the same skill. (Derek, age 33, Male, F-1 visa)

Even students experienced discrimination based on their race and ethnicity in academic settings, particularly in environments where the majority of students belonged to the White community. Michael shared his experience of feeling discriminated against while he was in Canada:

When I was in Canada… . all the white people, the Canadians… were kind of teasing me… . It's not very distinguishable but… they're making fun of me. (Michael, age 35, Male, F-1 visa).

##### 3.3.2.2 Underestimation

Immigrants experience the challenges of facing underestimation due to their race and ethnicity. The pre-existing assumption that one race is more competent than another had affected several participants at their workplace. Clara worked as a front desk supervisor at a hotel in Las Vegas with two White co-workers who were both new workers. She shared her experience of a family assuming that the White worker would be more capable and knowledgeable than her just because of the difference in race:

The family went to the White person, but because she's [(the co-worker)] new, she didn't know how to find the room so she called me for help… . They [(the family)] kind of had a look like, “Why is this white person asking an Asian person?” They think [the] White person can do it better than [an] Asian… . They just automatically assume. (Clara, age 46, Female, B-1 or B-2 visa)

##### 3.3.2.3 Outcasted/segregated

As immigrants move to the U.S., many face segregation due to ethnic disparities, leading to social isolation. Korean immigrants were not always welcomed by the local community and therefore, felt outcasted and segregated. Danielle shared her experience of ethnic discrimination during her stay in Florida. She mentioned that she was often directed to sit at the corner of the restaurants. Danielle felt as if the restaurant was trying to hide her because she was “different” from the local people:

In Florida, whenever I go to [a] restaurant, they sat us [at the] very corner and they never came to our table. I felt like they're hiding us from the front or other people. It's very subtle but I felt like it's discrimination. (Danielle, age 32, Female, F-1 visa)

In addition, Sarah shared her experience of facing discrimination due to her ethnicity. She felt outcasted for being one of the few Asian immigrants in Kansas. Particularly in states with predominantly White communities, immigrants often faced segregation due to their minority status. When Sarah walked into a restaurant, the customers frequently stared at her, making it uncomfortable for her to go out:

In Kansas, 90 plus percent is all White. When I walk into [a] Japanese restaurant, they [(the other customers)] [were] looking at me. (Sarah, age 42, Female, B-1 or B-2 visa)

##### 3.3.2.4 Name-calling

Asian immigrants often face racial discrimination through name-calling, which is more prevalent in states where Asians are the minority. Many participants shared their discomfort of feeling insulted from being victims of name-calling. Yvonne encountered an incident in New York where a random worker referred to her as “yellow” while she was driving:

Some guy call me yellow… . He was working outside. I didn't know him. (Yvonne, age 44, Female, J-1 visa)

Donald experienced a similar situation in Southern California when two kids called him “Ching chong” at a grocery store. “Ching chong” is an ethnic slur typically used to imitate Chinese language with an offensive connotation:

I was going to a store and coming out and two Caucasian kids said “Ching chong” as they moved past me. (Donald, age 35, Male, U.S. citizenship)

##### 3.3.2.5 Verbal insult

Korean immigrants experienced verbal insults regarding their immigrant status. Especially in predominantly White communities, immigrants were often viewed as outsiders and unwelcomed in the U.S. Immigrants were frequently told to leave the U.S. and return back to their home country. Yasmine encountered a group of students telling her to “go back” to Korea. While most of the students at her boarding school were Asians, some immigrants were insulted by the local students:

When we [passed] by some group of White students, [they] would be like “Go! Go back to your country.” (Yasmine, age 25, Female, F-1 visa)

In addition, Jeremy shared similar experiences of feeling unwelcomed in his new neighborhood after moving to the U.S., as his neighbors verbally excluded him from the community. He further mentioned that discrimination existed everywhere, especially among people from different backgrounds than his own:

No matter where it is, they'll be like, “Hey, why did you come here? You're an outsider. This is for… the neighborhood people only, you know.” (Jeremy, age 32, Male, F-2 visa)

##### 3.3.2.6 Physical assault

In addition to name-calling and verbal insults, immigrants were occasionally physically assaulted. Lisa shared her experience of witnessing Asian immigrants being victims of physical attacks after leaving her home country, Korea. Lisa expressed her shock to see local people throw canned beer and fireworks to the Asian immigrants:

They [(White people)] threw beer cans to the Asian women and threw some fireworks to Asian people. (Lisa, age 33, Female, F-1 visa)

#### 3.3.3 Cultural differences

##### 3.3.3.1 Devaluing Korean culture

As immigrants moved to the U.S., many faced discrimination due to cultural differences. Several immigrants experienced cultural discrimination through the devaluation of their Korean culture. While shopping for a dress in Ohio to attend a friend's wedding, Jessica mentioned that the workers displayed a condescending attitude toward Korean culture. She felt hurt after hearing someone talk down on her culture and country:

I went to [Clothing Store] to buy [a] dress… . They [said], “I bet they don't have nice clothes like this there [(Jessica's home country, Korea)]” (Jessica, age 32, Female, U.S. citizenship)

##### 3.3.3.2 Insulting Korean cuisine/clothing

Due to ethnic differences, immigrants experienced insults toward Korean cuisine. Particularly for those living in predominantly White communities, such as Jessica, Korean immigrants experienced offensive insults. While working in Ohio, Jessica shared that her coworkers had made fun of her lunch. She expressed her hurt feelings and shock as the other coworkers laughed along:

When I was working at [Automobile Company], I packed [a] Korean lunch. And my coworker asked me, “What is the nasty shit are you're eating?… . It's nasty. You call that food?”. (Jessica, age 32, Female, U.S. citizenship)

Furthermore, Sienna shared her experience of cultural discrimination through the insult of her clothing. At the U.S. customs interview, Sienna mentioned that a Jamaican officer liked her nails and dress. However, the officer gave the impression of not wanting to deal with her due to her cultural background. She felt as if the officer looked down on her because she was an Asian immigrant. If Sienna were of the same ethnicity as the officer, she mentioned that the officer would have most likely treated her with more respect:

At the customs [(U.S. customs)] interview, the… Jamaican lady… looked [at] me up and down and then just didn't want to deal with me… . . If I looked exactly like her, then I think she would have been more graceful… more merciful. (Sienna, age 36, Female, F-1 visa)

### 3.4 Many times, immigrant's mental expectations and reality of coming to the U.S. were inconsistent, affecting their psychological health as they attempted to adjust to the new environment

Immigrants struggled to adapt to the new environment as they experienced inconsistencies between their expectations and the reality of coming to the U.S. According to Berry's acculturation model, psychological well-being is influenced by acculturative stress, which is associated with adapting to the host society (Berry, 2017). Likewise, Portes and Zhou's Segmented Assimilation Theory suggests that acculturative stress may vary depending on their social environments (Portes and Zhou, 1993). Participants faced culture shock due to the differences between Korean and American culture. Immigrants generally shared their experiences of discomfort during the initial months following their stay in the U.S. The factors impacting their psychological well-being have been examined and listed below.

#### 3.4.1 Expectation vs. reality

##### 3.4.1.1 Ethnic diversity

Korean immigrants generally expected the U.S. to be ethnically and socially diverse. For immigrants residing in ethnically diverse states including California, which is the highest state in the U.S. for Korean immigrants to stay in 2022 (Esterline and Batalova, 2022), their expectations and reality were consistent. However, for immigrants such as Jeffery who moved to states that are considered predominantly White, their expectations and realities were not met. Jeffery shared his struggle of making friends and fitting into the community as his school was not as ethnically diverse as he expected:

There were maybe five Asians in [my] middle school. It's [a] pretty diverse school, but [there were] not many Asians. So [I] couldn't make friends. That was pretty tough. (Jeffery, age 35, Male, F-1 visa)

##### 3.4.1.2 Individualism/collectivism

The cultural differences between the Korean collectivist background and the U.S. individualistic background were identified by the Korean immigrants. Several participants described people in the U.S. as “very opinionated” and having their own voice, which was respected by others. In contrast, Joy emphasized the concept of “major” and “minor” in Korea, where social hierarchies discouraged individuals from speaking out. She explained that being a “major” is seen as superior to being a “minor.” Because of the social segregation, individuals in Korea tend to conform to the majority rather than standing out and having a voice. The emphasis of individualism in the U.S. required participants to shift from a collectivist mindset to a culture that values independence and self-expression. By adapting to an assertive communication style, many Korean immigrants reported feeling more valued and respected:

[In] Korea, there's major and there's minor. Minor doesn't speak much… . More people were listening and accepting [in the U.S.]… That's why people here feel like they're okay to express more about what they want, what they don't want. And then expect somebody to respect that voice. (Joy, age 34, Female, Green card)

#### 3.4.2 Culture shock

##### 3.4.2.1 Respect toward elders

The interaction with elders, specifically the differences in level of respect, had been a cultural difference experienced by Korean immigrants. Typically, participants described Korea as having a vertical relationship, especially within the workplace, where individuals with higher status are expected to be highly respected. On the other hand, they described the U.S. as having a more horizontal relationship where individuals are generally treated with equal respect. This cultural shift required participants to adapt to the American egalitarian culture, which emphasizes equality in the workplace and interpersonal relationships. While some participants initially struggled to adjust, many shared that the horizontal relationship allowed them to feel more included and valued in professional settings. When first moving to the U.S., Clay described his feeling of discomfort when calling his manager by his first name. He explained the Korean cultural expectation for individuals to address elder people with respect (not by their first names):

I feel uncomfortable treating older person, like my manager. When you meet your manager, you just say “Hi,” and their name. [If] their name is Kevin. “Hi, Kevin.” We don't do that in Korea. So I felt very uncomfortable with that at the beginning. [The] relationship between people is very different. (Clay, age 30, Male, J-1 visa)

##### 3.4.2.2 Friendly strangers

According to Korean immigrants, individuals are less reserved and more friendly in the U.S. culture. Participants described that individuals tend to be less sociable to strangers and not engage in conversations with others. Clay shared his experience of encountering strangers in an elevator that tried to start conversations with him. Initially, he felt uncomfortable and struggled to adjust to the U.S. culture:

Small talks. When you're in [an] elevator and someone comes in, they ask me something, and I feel awkward. I'm not getting used to it at all… . It's [a] very friendly culture but I don't like talking with strangers. (Clay, age 30, Male, J-1 visa)

##### 3.4.2.3 LGBTQ community

In contrast to Asian countries, immigrants felt that the U.S. places a stronger emphasis on the LGBTQ community. As a Korean immigrant, Michael was not familiar with the concept of being “gay” or “lesbian” until he came to the U.S. He shared how people in Korea were more likely to conform with the community to prevent standing out. Therefore, Michael was shocked with how individuals were able to freely express themselves in the U.S.:

I saw a gay… That wasn't in my dictionary. All that concept… . They were proud to be gay. They're saying, “I'm gay or I'm lesbian”. That was kind of shocking to me at first. (Michael, age 35, Male, U.S. citizenship)

##### 3.4.2.4 Homeless population

Korean immigrants showed concerns about the homeless population in the U.S. Several participants expressed feelings of insecurity living in San Francisco due to seeing many homeless and smelling weed in the streets. Furthermore, Clay shared how people were more likely to get involved with drugs in the U.S. Back in Korea, he had not been closely exposed to the homeless or to drugs:

There are so many homeless in the San Francisco… . I smell weed every day in the street so I felt very insecure. (Clay, age 30, Male, J-1 visa)

##### 3.4.2.5 Gun control

Negative initial expectations of the U.S. were often concerns related to the U.S. gun control policies. Many participants perceived the U.S. to be dangerous and felt insecure going to work in the first few weeks of coming to the U.S. Clay mentioned frequently seeing the U.S. news on gun violence, which was inconceivable to happen in Korea. His family expressed signs of hesitancy regarding his decision to move to the U.S. as they were concerned with the dangers of gun use:

I felt like U.S. is [a] very dangerous place because gun is legal… . [At the] beginning of my internship in [my] first year, I felt very insecure everyday. (Clay, age 30, Male, J-1 visa)

Wesley shared similar initial expectations of U.S. being a dangerous country due to the freedom to own and use guns. He further mentioned that his father was hesitant to let him move to the U.S. because he believed Americans were dangerous, which were shaped from watching American movies:

In the same way my dad was… very hesitant to come to [the] States because of the movies he watched. He was set in mind that Americans all have guns and it's dangerous to come. (Wesley, age 20, Male, F-4 visa)

## 4 Discussion

### 4.1 Legal status transitions and associated challenges

This study investigates the challenges Korean immigrants in the U.S. face when changing legal status, including their experiences of racism or discrimination and difficulties with adjusting to the new environment. Furthermore, we analyze whether the difficulties immigrants experience are different between obtaining primary legal status when moving to the U.S. and when obtaining current legal status after moving to the U.S. As immigrants attempt to obtain their current visa, they experience an additional set of challenges through racism or discrimination and cultural shocks after moving to the U.S. The purpose of this study is to compare the difficulties associated with obtaining primary legal status and the challenges of obtaining current legal status. Additionally, this study examines the impact of changes in legal status on the mental well-being of Korean immigrants.

### 4.2 Challenges in visa transition and sponsorship

A significant percentage of immigrants in the U.S. are international students, who later transition to become permanent residents, due to the economic and professional advantages of staying in the U.S. (Hailu et al., 2012; Hazen and Alberts, 2006; Pillai et al., 2024). Immigrants generally came to the U.S. with their families on an F-1 student visa and currently hold an H1-B visa, green card, or U.S. citizenship. Issues related to visas before moving to the U.S. were more commonly associated with family visa conflicts as the application for an F-1 student visa had been managed by the immigrant's primary school in Korea. Participants commonly experienced more challenges transitioning to their current legal status compared to when obtaining their primary legal status. After moving to the U.S., immigrants applying for an H1-B visa, green card, or U.S. citizenship emphasized the transition of difficulties from external factors (such as dealing with family visa conflicts) to internal factors (such as overcoming pressure and competition). After completing their college education, immigrants encountered difficulties in obtaining a working visa or green card through the visa lottery system or company sponsorship to stay in the U.S. Especially when obtaining an H1-B visa, immigrants highlighted the need for patience as they waited to be selected from the visa lottery and alertness as they had to meet visa expiration deadlines while waiting for approval for renewal or change of their visa status. Immigrants particularly determined to remain in the U.S. felt the pressure to demonstrate their value within the company in order to be sponsored for their green card visa. They emphasized the importance of competitiveness as the internal pressure to “prove” themselves was a motivation to work hard within the company.

### 4.3 Cultural adjustment, discrimination, and mental health impacts

Participants experienced acculturation challenges as their collectivist values and emphasis on hierarchical respect intersect with the individualistic and egalitarian culture of the U.S. This clash could lead to cultural dissonance, impacting communication styles, workplace interactions, and social integration. Triandis (2018) highlights how individualism emphasizes personal autonomy, while collectivist cultures from East Asian countries including Korea prioritize group harmony and familial obligations. Santos and Kawabata (2023) found that higher levels of social connectedness and enculturation (maintaining cultural identity) were associated with better mental health outcomes, while acculturative stress negatively impacted mental health among Asian American and Pacific Islander immigrants. Social connectedness served as a protective factor, buffering against the adverse effects of acculturative stress. Immigrants expressed their experiences of unease and discomfort in the first few months during the initial months following their arrival in the U.S. (Hailu et al., 2012; Jimenez, 2011). Cultural differences in the workplace have especially affected immigrants coming to the U.S. with an H1-B visa due to the vertical and horizontal relationship differences and variations in conversational interactions between Korea and the U.S. Upon moving to the U.S., immigrants of minorities often encounter racism and discrimination due to their cultural disparities (Chung and Epstein, 2014; Pillai et al., 2024). Among the participants, these measures of discrimination were influenced by whether they had previously resided in another country before coming to the U.S. and the immigrants' state of residence within the U.S. Immigrants who studied in another country (e.g. Canada or the UK) before moving to the U.S. were less prone to facing challenges in adapting to American culture, as opposed to those who directly immigrated to the U.S. Moreover, the findings support research indicating the significance of nature in immigrants' ability to adapt to the host country (Gibson, 2001; Hurh and Kim, 1984; Stodolska et al., 2017). Participants who lived in predominantly White states found it harder to adapt to the new environment and experienced more discrimination compared to those who moved to a predominantly Asian community. Language barriers and cultural differences were prominent factors contributing to discrimination, especially among individuals residing in White communities. Immigrants living in predominantly White populations encountered greater disparities in their mental expectations of the U.S., resulting in challenges adapting to the American community. Overall, research indicates that immigrants frequently encounter a disconnect between their expectations and the reality of living in the U.S. (Hailu et al., 2012; Noe-Bustamante et al., 2022). Difficulties in communication, adjusting to cultural differences, and facing racial discrimination created inconsistencies between the immigrants' mental expectations of the U.S., leading to mental health concerns including anxiety and insecurity. Anxiety and insecurity often persist as issues for immigrants, as they continuously experience fear of discrimination, challenges in communication, and acculturative stress. Over time, these feelings can lead to self-doubt and a diminished sense of self-esteem, affecting other areas of life including social connections and career opportunities, further interfering with their ability to adapt to the community.

### 4.4 Strengths and limitations

This study provides valuable insights into the experiences of Korean immigrants, addressing a significant gap in the literature by focusing on the intersection of legal status transitions and mental health. Through rich qualitative data from in-depth interviews, the study captures the personal narratives and nuanced challenges faced by immigrants, offering perspectives that quantitative methods may overlook. The community-centered approach, involving participants from a Korean Christian church, fosters trust and facilitates open discussions, enhancing the depth of the data collected. By examining the psychological impact of visa transitions and experiences of discrimination, the study contributes to a more comprehensive understanding of immigrant well-being. Furthermore, the findings hold practical implications for immigration policy, highlighting the need for reforms to better support immigrants' mental health during periods of legal uncertainty and transition.

However, there are potential limitations. This study was the selection of participants exclusively from a Korean Christian church. This recruitment criterion was implemented to facilitate access to a cohesive immigrant community and ensure participant willingness to engage in-depth interviews. However, this decision may introduce bias, as participants' experiences and perspectives could be shaped by shared religious and cultural values. We acknowledge that the views expressed by participants may not fully represent the broader Korean immigrant population, particularly those from non-religious or more secular backgrounds. This homogeneity in the sample may have affected the range of responses, potentially underreporting more liberal perspectives on social issues such as drug use or LGBTQ rights. Another limitation lies in the data collection process, as interviews were conducted by multiple researchers involved in the study. While this collaborative approach allowed for the inclusion of diverse perspectives and facilitated efficient data gathering, it may have introduced inconsistencies in how questions were framed or followed up. The semi-structured nature of the interviews, while advantageous for allowing participants to elaborate on their experiences, also left room for variability in the depth and focus of responses depending on the interviewer. Differences in tone, phrasing, and probing techniques across researchers could have influenced participant disclosures, potentially impacting the uniformity of data collected. To mitigate this, the research team held regular debriefing sessions to align on key interview techniques and review the overarching goals of the interviews. However, we recognize that subtle variations in interviewer styles are inevitable.

Future studies should consider a multifaceted approach that addresses both methodological consistency and broader participant diversity. Standardized training protocols or pilot interviews could help ensure uniformity in the data collection process, while designating a single primary interviewer or conducting post-interview debriefs may reduce variability across sessions. Additionally, expanding recruitment efforts to engage Korean immigrants from secular organizations, non-religious groups, and diverse social networks would provide a broader range of perspectives, mitigating biases linked to religious affiliation. Incorporating intersectional factors such as gender, age, and socioeconomic status could further enrich the analysis, allowing for a deeper understanding of how overlapping identities shape the immigrant experience. This comprehensive approach would enhance the generalizability of findings and contribute to more inclusive research that informs policies and interventions aimed at addressing the diverse needs of immigrant communities.

## 5 Conclusion

Immigrants experience greater difficulties in obtaining and maintaining their legal status after arriving in the U.S. Furthermore, the geographical location within the U.S. to which immigrants migrated contributes to the varying levels of racism and discrimination experienced, as well as the challenges in adjusting to the new environment. Immigrants encounter distinct challenges when changing their legal status. By including the psychological impact of obtaining varying legal statuses among Korean immigrants, this study sheds new light on the difficulties encountered during the transition from one legal status to another, both before and after coming to the U.S. It is important to offer a new perspective on the challenges faced by immigrants by examining the differences in obtaining their initial legal status before coming to the U.S. and transitioning to their current legal status after arriving in the U.S. By expanding the immigrant group to include various countries of origin, future studies can evaluate the impact of changes in legal status on the psychological well-being of immigrants through an examination of their experiences of racism and discrimination and an analysis of the inconsistencies between their expectations and reality.

## Ethics approval and consent to participate

The institutional review board at San José State University approved this study (IRB Protocol Tracking Number: 23026). Informed consent was obtained from all participants at the beginning of the online survey. Subsequently, consent forms with the required signatures were collected at the start of the in-depth interviews.

## Availability of data and materials

The datasets generated and/or analyzed during the current study are not publicly available due to participant confidentiality but are available from the corresponding author on reasonable request.

## Funding

Research reported in this publication was supported by the Division of Research and Innovation at San José State University under Award Number 23-RSG-05-061 and by the National Institute of General Medical Sciences of the National Institutes of Health under Award Number R16GM150715. The content is solely the responsibility of the authors and does not necessarily represent the official views of San José State University and the National Institutes of Health.

## CRediT authorship contribution statement

**Chulwoo Park:** Writing – review & editing, Validation, Supervision, Resources, Project administration, Methodology, Investigation, Funding acquisition, Formal analysis, Data curation, Conceptualization. **Airi Irene Trisnadi:** Writing – original draft, Visualization, Investigation, Formal analysis, Data curation.

## Declaration of competing interest

The authors declare that they have no competing interests.

## References

[bib0001] Alegría M., Álvarez K., DiMarzio K. (2017). Immigration and Mental Health. Curr. Epidemiol. Rep..

[bib0002] Berry J.W. (2017). Theories and models of acculturation. Oxf. Handb. Accult. Health.

[bib0003] Berry J.W. (1997). Immigration, acculturation, and adaptation. Appl. Psychol..

[bib0004] Brabeck K.M., Sibley E. (2016). Immigrant parent legal status, parent–Child relationships, and child social emotional wellbeing: a middle childhood perspective. J. Child Fam. Stud..

[bib0005] Cho Y.J., Lee W.J., Oh H., Lee J.O., Kim B.-K.E., Jang Y. (2022). Perceived racial discrimination and mental health in diverse groups of Asian Americans: the differing impacts by age, education, and ethnicity. J. Immigr. Minor. Health.

[bib0006] Chung H., Epstein N.B. (2014). Perceived racial discrimination, acculturative stress, and psychological distress among Asian immigrants: the moderating effects of support and interpersonal strain from a partner. Int. J. Intercult. Relat..

[bib0007] Esterline, C., Batalova, J., 2022. Korean Immigrants in the United States [WWW Document]. migrationpolicy.org. URL https://www.migrationpolicy.org/article/korean-immigrants-united-states (accessed 8.16.24).

[bib0008] Gee G.C., Ford C.L. (2011). Structural racism and health inequities: old issues, new Directions1. Bois Rev. Soc. Sci. Res. Race.

[bib0009] Gelatt, J., 2017. The RAISE Act: Dramatic Change to Family Immigration, Less So for the Employment-Based System [WWW Document]. migrationpolicy.org. URL https://www.migrationpolicy.org/news/raise-act-dramatic-change-family-immigration-less-so-employment-based-system (accessed 7.11.24).

[bib0010] Gibson M.A. (2001). Immigrant adaptation and patterns of acculturation. Hum. Dev..

[bib0011] Hailu T.E., Mendoza B.M., Lahman M.K.E., Richard V.M. (2012). Lived experiences of diversity visa lottery immigrants in the United States. Qual. Rep..

[bib0012] Hazen H.D., Alberts H.C. (2006). Visitors or immigrants? International students in the United States. Popul. Space Place.

[bib0013] Hurh W.M., Kim K.C. (1984). Adhesive sociocultural adaptation of Korean immigrants in the U.S.: an alternative strategy of minority adaptation. Int. Migr. Rev..

[bib0014] Jacobs E.M. (2019). Pathways to permanence: legal status transitions as a key mechanism in skilled migrant selection and settlement. Front. Sociol..

[bib0015] Jimenez, T.R., 2011. Immigrants in the United States: how well are they integrating into society?

[bib0016] Kim J. (2022). A case study of international students' Challenges. KJECTL.

[bib0017] Massey D.S., Arango J., Hugo G., Kouaouci A., Pellegrino A., Taylor J.E. (1993). Theories of International migration: a review and appraisal. Popul. Dev. Rev..

[bib0018] Nadimpalli S.B., Kanaya A.M., McDade T.W., Kandula N.R. (2016). Self-reported discrimination and mental health among Asian Indians: cultural beliefs and coping style as moderators. Asian Am. J. Psychol..

[bib0019] Noe-Bustamante, L., Mora, L., Ruiz, N.G., 2022. Where Asian immigrants face language challenges: navigating daily life and communicating in English [WWW Document]. Pew Res. Cent. URL https://www.pewresearch.org/2022/12/19/where-asian-immigrants-face-language-challenges-a-anavigating-daily-life-and-communicating-in-english/ (accessed 7.11.24).

[bib0020] Office of Homeland Security Statistics, 2023. 2022 Yearbook of Immigration Statistics.

[bib0021] Pillai, D., Artiga, S., Hamel, L., Schumacher, S., Kirzinger, A., Rao, A., Published, A.K., 2024. Understanding the diversity in the Asian immigrant experience in the U.S.: the 2023 KFF/LA Times survey of Immigrants. KFF. URL https://www.kff.org/report-section/understanding-the-diversity-in-the-asian-immigrant-experience-in-the-u-s-findings/ (accessed 7.11.24).

[bib0022] Portes A., Zhou M. (1993). The new Second generation: segmented assimilation and its variants. Ann. Am. Acad. Pol. Soc. Sci..

[bib0023] Ruiz, N.G., Im, C., Tian, Z., 2023. Asian Americans' experiences with discrimination in their daily lives [WWW Document]. URL https://www.pewresearch.org/2023/11/30/asian-americans-experiences-with-discrimination-in-their-daily-lives/(accessed 7.11.24).

[bib0024] Santos J.M., Kawabata Y. (2023). A path model of acculturation, enculturation, social connectedness, and mental health among Asian American/Pacific Islander immigrants. J. Cross-Cult. Psychol..

[bib0025] Singh S., Schulz A.J., Neighbors H.W., Griffith D.M. (2017). Interactive effect of immigration-related factors with legal and discrimination acculturative stress in predicting depression among Asian American immigrants. Community Ment. Health J..

[bib0026] Stodolska M., Peters K., Horolets A. (2017). Immigrants' Adaptation and interracial/interethnic interactions in natural environments. Leis. Sci..

[bib0027] Triandis H.C. (2018).

[bib0028] U.S. Census Bureau, 2022. Selected characteristics of the foreign-born population by period of entry into the United States [WWW Document]. URL https://data.census.gov/table/ACSST1Y2022.S0502?q=immigrant (accessed 7.11.24).

[bib0029] U.S. Citizenship and Immigration Services, 2024a. H-1B Cap Season [WWW Document]. URL https://www.uscis.gov/working-in-the-united-states/temporary-workers/h-1b-specialty-occupations-and-fashion-models/h-1b-cap-season (accessed 8.16.24).

[bib0030] U.S. Citizenship and Immigration Services, 2024b H-1B specialty occupations, DOD Cooperative Research and Development Project workers, and fashion models [WWW Document]. URL https://www.uscis.gov/working-in-the-united-states/h-1b-specialty-occupations (accessed 7.11.24).

[bib0031] U.S. Citizenship and Immigration Services, 2023. Students and exchange visitors [WWW Document]. URL https://www.uscis.gov/working-in-the-united-states/students-and-exchange-visitors (accessed 7.11.24).

[bib0032] U.S. Citizenship and Immigration Services, 2022. Green Card for employment-based immigrants [WWW Document]. URL https://www.uscis.gov/green-card/green-card-eligibility/green-card-for-employment-based-immigrants (accessed 7.11.24).

[bib0033] U.S. Citizenship and Immigration Services, 2020a. Citizenship and naturalization [WWW Document]. URL https://www.uscis.gov/citizenship/learn-about-citizenship/citizenship-and-naturalization (accessed 11.8.24).

[bib0034] U.S. Citizenship and Immigration Services, 2020b COVID-19 delays in extension/change of status filings [WWW Document]. URL https://www.uscis.gov/archive/covid-19-delays-in-extensionchange-of-status-filings (accessed 7.11.24).

[bib0035] U.S. Department of Commerce, 2023. U.S. Census Bureau releases key stats in honor of 2023 asian American, native Hawaiian, and Pacific Islander Heritage month [WWW Document]. US Dep. Commer. URL https://www.commerce.gov/news/blog/2023/05/us-census-bureau-releases-key-stats-honor-2023-asian-american-native-hawaiian-and (accessed 7.11.24).

[bib0036] U.S. Department of Homeland Security, 2023. Legal Immigration and Adjustment of Status Report Fiscal year 2023, quarter 4 [WWW Document]. URL https://www.dhs.gov/immigration-statistics/special-reports/legal-immigration (accessed 7.11.24).

[bib0037] U.S. Department of Homeland Security, 2022. Profiles on naturalized citizens: 2022 country [WWW Document]. URL https://www.dhs.gov/ohss/topics/immigration/naturalizations/profiles/2022/country (accessed 7.11.24).

[bib0038] U.S. Department of State - Bureau of Consular Affairs, 2024. Immigrate [WWW Document]. URL https://travel.state.gov/content/travel/en/us-visas/immigrate.html (accessed 7.11.24).

[bib0039] Yoo H.C., Gee G.C., Takeuchi D. (2009). Discrimination and health among Asian American immigrants: disentangling racial from language discrimination. Soc. Sci. Med..

